# Neurophenotypes of COVID-19: risk factors and recovery outcomes

**DOI:** 10.21203/rs.3.rs-2363210/v2

**Published:** 2023-02-20

**Authors:** Divya Prabhakaran, Gregory S. Day, Bala Munipalli, Beth K. Rush, Lauren Pudalov, Shehzad K. Niazi, Emily Brennan, Harry R. Powers, Ravi Durvasula, Arjun Athreya, Karen Blackmon

**Affiliations:** Mayo Clinic; Mayo Clinic; Mayo Clinic; Mayo Clinic; Mayo Clinic; Mayo Clinic; Mayo Clinic; Mayo Clinic; Mayo Clinic

**Keywords:** COVID-19, long COVID, post-acute sequelae of COVID-19, area deprivation index, neuropsychology

## Abstract

Coronavirus disease 2019 (COVID-19) infection is associated with risk of persistent neurocognitive and neuropsychiatric complications, termed “long COVID”. It is unclear whether the neuropsychological manifestations of COVID-19 present as a uniform syndrome or as distinct neurophenotypes with differing risk factors and recovery outcomes. We examined post-acute neuropsychological profiles following SARS-CoV-2 infection in 205 patients recruited from inpatient and outpatient populations, using an unsupervised machine learning cluster analysis, with objective and subjective measures as input features. This resulted in three distinct post-COVID clusters. In the largest cluster (69%), cognitive functions were within normal limits, although mild subjective attention and memory complaints were reported. Vaccination was associated with membership in this “normal cognition” phenotype. Cognitive impairment was present in the remaining 31% of the sample but clustered into two differentially impaired groups. In 16% of participants, memory deficits, slowed processing speed, and fatigue were predominant. Risk factors for membership in the “memory-speed impaired” neurophenotype included anosmia and more severe COVID-19 infection. In the remaining 15% of participants, executive dysfunction was predominant. Risk factors for membership in this milder “dysexecutive” neurophenotype included disease-nonspecific factors such as neighborhood deprivation and obesity. Recovery outcomes at 6-month follow-up differed across neurophenotypes, with the normal cognition group showing improvement in verbal memory and psychomotor speed, the dysexecutive group showing improvement in cognitive flexibility, and the memory-speed impaired group showing no objective improvement and relatively worse functional outcomes compared to the other two clusters. These results indicate that there are multiple post-acute neurophenotypes of long COVID, with different etiological pathways and recovery outcomes. This information may inform phenotype-specific approaches to treatment.

## Introduction

Cognitive and psychiatric symptoms are among the most common, persistent, and disabling consequences of COVID-19 (Taquet et al., 2022; Hastie et al., 2022; Davis et al., 2021). Post-COVID cognitive impairment is reported in 15-35% of patients during the chronic “long COVID” recovery phase (Ceban et al., 2022; Becker et al., 2021), with higher rates reported in patients who were hospitalized versus home-isolated during the acute stage of illness (Pihlaja et al., 2023). Self-reported cognitive complaints include problems with concentration, memory, and slowed thinking (Davis et al., 2021; Ceban et al., 2022), commonly referred to as “brain fog”. Objective neuropsychological assessment shows predominant impairment in attention, executive functioning, and memory, with relative preservation of language and visuospatial functions (Bertuccelli et al., 2022). The most common post-COVID neuropsychiatric manifestations (new onset and chronic) include fatigue, anxiety, depression, insomnia, and posttraumatic stress disorder (PTSD) (Nalbandian et al., 2021). At this point, it remains unclear whether these heterogenous cognitive and psychiatric sequelae are uniformly elevated or clustered into distinct post-COVID neurophenotypes. This is important to determine, as different post-acute neurophenotypic presentations could point backwards to different etiological factors and forwards to different recovery outcomes.

Several mechanisms may contribute to persistent cognitive and psychiatric sequelae following COVID-19 infection. These include neuroinvasion of SARS-CoV-2 into brain or neuroepithelial tissue and indirect damage from respiratory failure (hypoxic-ischemic effects), stroke, multi-organ system dysfunction, inflammasome activation, and complex pandemic-related psychosocial factors, such as social isolation, fear, and altered sleep, diet, exercise, and other health behaviors (Crunfli et al., 2022; Mehandru and Merad, 2022; Evans et al., 2021; Ollila et al., 2022). Risk factors for long COVID are heterogeneous. Higher COVID-19 disease severity is associated with greater risk of long COVID (Shir and Day, 2022) but even mild to moderate infections can increase risk of adverse neuropsychiatric outcomes (Taquet et al., 2022; Zhao et al., 2022). Cognitive profiles vary across studies, and it remains unclear whether disease-specific features (i.e., acute COVID-19 severity, presence of anosmia, encephalopathy, stroke, etc.) and disease-nonspecific features (sociodemographics, medical comorbidities, etc.) differentially contribute to distinct post-acute neuropsychological profiles. Given the variety of explanatory factors and ways in which they may interact, it is likely that the neuropsychological manifestations of long COVID present as a multiform rather than a uniform cognitive syndrome.

Recognizing this, we examined objective and subjective neuropsychological outcomes of SARS-CoV-2 infection in ambulatory and hospitalized patients using standardized assessment procedures across successive COVID-19 variant waves. We characterized multivariate neuropsychological clusters (i.e., “neurophenotypes”) during the post-acute recovery stage, identified their associative features, and re-examined recovery outcomes six months later with the same symptom inventories and computerized cognitive testing platform. We hypothesized that neurocognitive and neuropsychiatric sequelae of COVID-19 would cluster into distinct neurophenotypes, each related to unique disease-specific and non-specific factors, and each differing in their longitudinal recovery trajectories.

## Materials And Methods

### 2.1 Participants

The Mayo Clinic Institutional Review Board approved this prospective longitudinal cohort study. We electronically obtained informed consent from all participants, recruited between July 2020 and February 2022 from a hospital-wide registry of Mayo Clinic Florida patients who tested positive for SARS-CoV-2 infection. All participants were ≥18 years of age and had no history of major neurocognitive disorders. Participants completed the outcome assessment within 12 weeks of PCR-confirmed infection (post-acute recovery stage) and follow-up assessments six months later (chronic recovery stage). Participation required access to a computer for consent, test, and survey completion. All participants received a digital link to complete assessments and respond to questionnaires.

We abstracted participant demographics and medical history from the electronic health record (EHR) at time of initial post-acute outcome assessment. Medical comorbidities were summarized with the Elixhauser Van-Walraven Index (EVCI). The EVCI includes 31 common chronic medical conditions and was calculated from EHR data up to 1 year before the PCR-positive test date (van Walraven et al., 2009). Higher scores indicate a greater number of medical comorbidities. Vascular risk factors (history of smoking, diabetes, hypertension, and obesity) were identified by ICD-10 diagnostic codes in the patient’s EHR (Kloppenborg et al., 2008) and separately summarized due to the specialized role that vascular risk factors play in adverse COVID-19 outcomes (Taquet et al., 2021). Sociodemographic disadvantage was summarized with the 2019 Area Deprivation Index (ADI). ADI scores for each participant were retrieved from the University of Wisconsin-Madison’s Neighborhood Atlas (2019), which derives national percentile rankings of socioeconomic disadvantage at the (US) Census Block Group neighborhood level from 1 (least disadvantaged) to 100 (most disadvantaged) based on unemployment rates, poverty, education, and housing (Kind and Buckingham, 2018).

COVID-19 disease severity was determined by an infectious disease specialist (HRP), using the National Institute of Allergy and Infection Disease Ordinal Scale (NIAID-OS) (Dodd et al., 2020), with lower scores indicating higher illness severity. The following COVID-19 disease-specific factors were assessed: hospitalization status (ambulatory versus hospitalized), symptom status (symptomatic versus asymptomatic), and presence of anosmia (yes/no). Vaccination status was coded at three levels: vaccine not available (prior to FDA approval); unvaccinated (vaccine FDA approved but participant remained unvaccinated); and vaccinated. Finally, COVID-19 variant type was estimated from peak variant prevalence data at covariants.org (Hodcroft, 2021) by test region (Florida) and time span (binned in 2-week intervals).

### 2.2. Neurocognitive Assessment

We assessed objective cognitive performance with the CNS-Vital Signs (CNSVS) computerized neurocognitive assessment during the post-acute and chronic stages of recovery. The CNSVS includes the following neurocognitive domains: (a) verbal memory (immediate and delayed word recognition); (b) visual memory (immediate and delayed design recognition); (c) psychomotor speed (finger tapping and symbol digit coding tests); (d) reaction time (averaged across Stroop congruent and incongruent trials); (e) complex attention (sum of errors from continuous performance, shifting attention, and Stroop tests); and (f) cognitive flexibility (correct responses on the shifting attention test minus the number of errors on the shifting attention test and Stroop test), as previously described (Gualtieri and Johnson, 2006). Domain scores were age-adjusted by comparison to a normative reference group (mean = 100, standard deviation = 15), which was collected by the test publisher prior to the COVID-pandemic (Gualtieri and Johnson, 2006). Classification of impairment (< 9th percentile) was based on the American Academy of Clinical Neuropsychology consensus conference statement on uniform labeling of performance test scores (Guilmette et al., 2020). CNSVS includes embedded validity indicators, which show overall high accuracy in identifying intentional attempts to underperform (Anderson et al., 2020). Scores that were flagged as invalid were removed before analyses.

### 2.3. Neuropsychiatric Symptom Inventories

We assessed subjective neuropsychological symptoms with the Neuropsych Questionnaire-45 (NPQ-45) during the post-acute and chronic stages of recovery. The NPQ-45 is a self-report symptom inventory that probes 12 neuropsychiatric symptom domains (Gualtieri, 2007). Scores from the following domains were summed and scaled as minimal (0-74), mild (75-149), or moderate to severe (150-300): attention (e.g., concentration difficulties), memory (e.g., forgetfulness), anxiety (e.g., nervousness, restlessness), depression (e.g., feeling discouraged, lack of interest), fatigue (e.g., low energy, weakness), and pain (e.g., headaches, muscle pain). During the chronic recovery stage, participants also completed the Medical Outcomes Survey (MOS-SF 36) (Ware and Sherbourne, 1992) and a posttraumatic stress disorder (PTSD) checklist (PCL-C 17) (Weathers et al., 1993).

### 2.4. Data Imputation

Multivariate Imputation via Chained Equations (MICE) with predictive mean matching for five imputations and 50 iterations (van Buuren and Groothuis-Oudshoorn, 2011) was used to complete missing data from the post-acute neurocognitive assessment and neuropsychiatric symptom inventories, with a <15% missingness threshold (Jakobsen et al., 2017). Little's test statistic was used to assess whether data was missing completely at random (MCAR) between cognitive domains (Little, 1988). Pearson χ2 and analysis of variance (ANOVA) statistical tests for parametric data and Kruskal-Wallis tests for non-parametric data were used to assess clinical and sociodemographic factors associated with cognitive domain data completeness.

### 2.5. Unsupervised Machine Learning: K-means Clustering

Unsupervised machine learning methods were used to perform cluster analyses, given that they allow for the inference of subgroups (referred to as clusters) within a dataset. Algorithms in unsupervised learning strive to maximize inter-cluster separation and minimize separation among samples within a cluster. Objective and subjective neuropsychological measures collected during the post-acute illness stage were used as input features in a K-means clustering analysis. No domain bias was applied to inputs. We determined the optimal number of k-means clusters with the elbow method (Kaufman, 1990). Model fitting was performed with NbClust (Charrad et al., 2014), implemented in R studio build 492 (Rstudio 2020), with R v4.1.1 (R Core Team, 2021). IBM SPSS Statistics 27 was used to perform K-means clustering and remaining data analyses. Pearson χ2 statistical test identified significant data missingness across clusters.

### 2.6. Cross-Sectional and Longitudinal Cluster Features

Clinical and sociodemographic factors associated with cluster membership were identified through Pearson χ2 and analysis of variance (ANOVA) statistical tests for parametric data and Kruskal-Wallis tests for non-parametric data. Normality was determined by kurtosis, skewness, and Shapiro-Wilk tests. Friedman non-parametric tests were used to evaluate longitudinal change in cognitive test scores and symptom inventory scores within each cluster. Analysis of variance (ANOVA) statistical tests were used to compare functional (MOS-36 scores) and psychiatric (PTSD scores) outcomes between clusters at the chronic (6-month) recovery stage. Significance was set at p < 0.05. Post-hoc analysis was conducted using Bonferroni correction to counteract Type I errors. Significance was set at p < 0.05.

## Results

During the study period, 205 participants (171 ambulatory, 34 hospitalized) completed post-acute neuropsychological outcome assessments 5.7 (± 3.8 weeks) following positive laboratory confirmation of SARS-CoV-2 infection. Of these, 101 participants completed the 6-month outcome assessment (87 ambulatory, 14 hospitalized). Attrition analyses are described in Supplementary Materials and Methods (Supplementary Figure 1 and Table 1).

### 3.1. Missing Data Analyses

The Little’s test result (test-statistic (29) = 20.97, p = 0.86) indicated that data was MCAR between cognitive domains. There were no differences associated with patient age, sex, education, ADI, hospitalization status, or NIAID score (p > 0.05) in cognitive domain data completeness. All input features met a <14% missingness threshold before MICE imputation.

### 3.2. Post-Acute Neuropsychological Outcomes K-means Cluster Analysis

**Figure 1:**
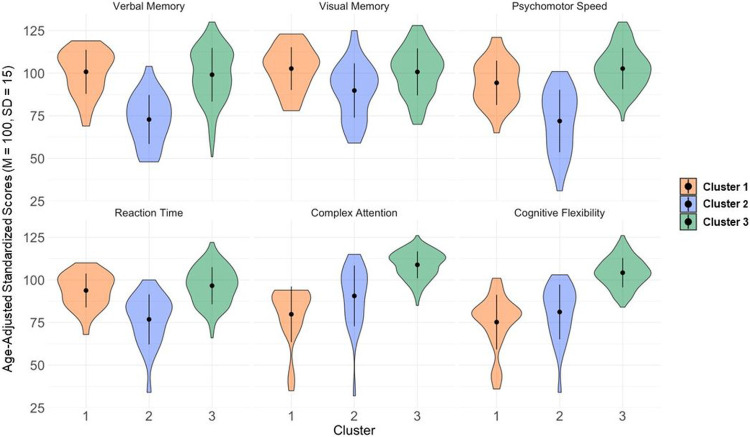
Violin plots show cluster centers for each cognitive domain score from the CNS Vital Signs computerized test battery. Scores were age-adjusted based on a normative reference sample with a mean of 100 and standard deviation of 15. Cluster 1 shows impaired cluster centers in complex attention and cognitive flexibility (dysexecutive group). Cluster 2 scores impaired cluster centers in verbal memory, psychomotor speed, and reaction time (memory-speed impaired group). Cluster 3 showed average/normal cluster centers for all cognitive domains (normal group).

We determined with the elbow method that the optimal number of clusters was k = 3. Clusters did not differ in data missingness (χ2 = 3.43, p = 0.22). Cluster 1 (N = 31) was characterized as “dysexecutive” due to impaired cluster centers for cognitive flexibility and complex attention (Figure 1, Supplementary Table 2). This dysexecutive cluster was also characterized by mild-to-moderate complaint severity for anxiety, attention, memory, fatigue, and pain. Cluster 2 (N = 32) was characterized as “memory-speed impaired” due to impaired cluster centers for verbal memory, psychomotor speed, and reaction time, as well as low average cluster centers for visual memory and cognitive flexibility. This memory-speed impaired cluster was also characterized by mild complaint severity for memory, attention, anxiety, depression, and pain, as well as moderate-severe fatigue. Cluster 3 was the largest cluster (N = 142) and was characterized as “normal cognition” due to cluster centers in the average/normal range for all cognitive domains. Notably, despite normal objective cognitive performance, participants in this cluster still reported mild complaint severity for attention, memory, fatigue, and pain.

To facilitate comparison of cognitive impairment rates with other studies reported in the literature, we calculated the rates of subjective and objective cognitive impairment by cluster and by domain in the post-acute recovery stage (Supplementary Table 3).

### 3.3. Disease-specific Risk Factors for Cluster Membership

There were no significant associations between cluster membership and COVID-19 variant type (χ2 = 5.56, p = 0.24) or symptom status (χ2 = 0.42, p = 0.81). There was a marginal relationship between cluster membership and hospitalization status (χ2 = 5.9, p = 0.05), with the highest hospitalization rates in the memory-speed impaired cluster (31%). Cluster membership was associated with vaccination status (χ2 = 11.64, p = 0.02); the normal cognition cluster had the highest vaccination rate (51%), while the memory-speed impaired cluster had the lowest vaccination rate (22%). Lack of vaccine availability at time of infection (i.e., infection before 12/17/20) was the most common reason why patients were unvaccinated across all 3 clusters. There was a strong association between cluster membership and anosmia (χ2 = 12.02, p = 0.002), as well as NIAID scores (H (2) = 10.20, p = 0.006), with the memory-speed impaired cluster showing the highest rate of anosmia (70%) and lowest median NIAID scores (higher disease severity). Full results are presented in Table 1.

### 3.4. Disease-nonspecific Risk factors for Cluster Membership

**Figure 2:**
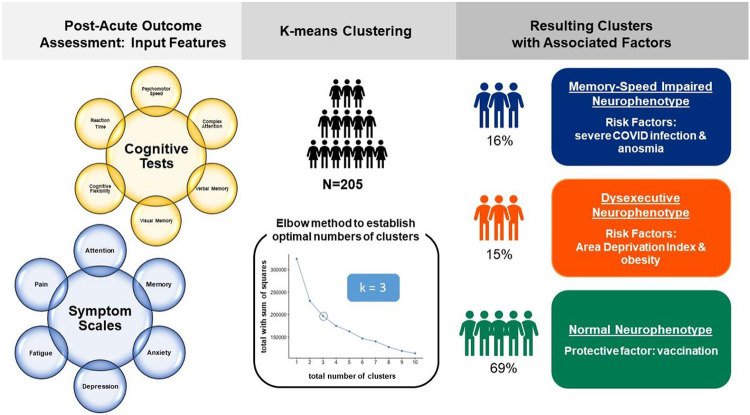
Clustering outcomes. Unsupervised machine learning methods were used to perform cluster analyses on input features collected during the post-acute recovery stage in 205 patients with PCR-confirmed COVID-19 infections. Patients ranged in illness severity from asymptomatic to mild (ambulatory) to severe (hospitalized) cases. The optimal number of k-means clusters was determined with the elbow method. The resulting clusters were found to be associated with different disease-specific (anosmia, illness severity) and disease-nonspecific (obesity and area deprivation) risk factors.

There were no differences in sex distribution (χ2 = 2.77, p = 0.25) or years of education (F = 0.78, p = 0.46) between clusters. Participant age differed across clusters (F = 8.25, p < 0.001), with the memory-speed impaired cluster showing the youngest mean age. Regarding medical risk factors, there were no associations between cluster membership and history of smoking (χ2 = 0.15, p = 0.93), hypertension (χ2 = 2.55, p = 0.28), diabetes (χ2 = 0.68, p = 0.71), or total EVCI score (H (2) = 0.68, p = 0.71). There was an association between cluster membership and obesity (χ2 = 8.82, p = 0.01) and ADI scores (H (2) = 6.49, p = 0.04). The dysexecutive cluster had the highest obesity rate (29%) while the normal cognition cluster had the lowest obesity rate (10%). ADI scores were highest in the dysexecutive cluster, indicating greater socioeconomic disadvantage. Summary results are depicted in Figure 2.

### 3.5. Longitudinal Recovery Outcomes

Of the original 205 participants who completed assessments in the post-acute recovery stage, 101 (49%) completed a follow-up assessment in the 6-month chronic recovery stage (dysexecutive cluster 1: N=11; memory-speed impaired cluster 2: N=12; normal cognition cluster 3: N=78). To facilitate comparison of chronic cognitive impairment rates with other studies reported in the literature, we calculated the rate of subjective and objective cognitive impairment by cluster and by domain in the chronic 6-month recovery stage (Supplementary Table 4). We compared impairment rates across clusters using chi-square analyses. Results show that cluster 1 (dysexecutive neurophenotype) no longer differs in rates of complex attention or cognitive flexibility impairment relative to the other 2 clusters at the 6-month recovery stage. The rate of verbal memory impairment rate in cluster 2 (memory-speed impaired neurophenotype) does not differ from the other clusters. However, cluster 2 does demonstrate higher rates of objective visual memory and psychomotor speed impairment at the 6-month recovery stage, as well as self-reports a higher rate of subjective memory impairment.

To examine within-subject change in objective performance and subjective symptoms over time, we used non-parametric Friedman test of differences among repeated measures. Results for each cluster are provided in Supplementary Table 5. Within cluster 1 (dysexecutive neurophenotype), there was marginal improvement in complex attention (p=0.06) and significant improvement in cognitive flexibility (p=0.01) but no change in subjective symptom report. Cluster 2 (memory-speed impaired neurophenotype) had no significant changes in objective cognitive test score, but they did self-report improved attention (p=0.03). Cluster 3 (“normal cognition” neurophenotype) significantly improved in the domains of verbal memory (p=0.01) and psychomotor speed (p=0.003) but had no change in subjective symptom inventory scores.

Comparison of functional outcomes between clusters at the 6-month chronic recovery stage revealed that cluster 2 (memory-speed impaired neurophenotype) had worse functional outcomes (MOS-36 scores) compared to the other two clusters (Table 2), with particularly strong effects for energy/fatigue, general health, and health change (i.e., decline in health relative to one year ago). There were no differences in PTSD PCL-17 scores between clusters.

## Discussion

In the current study, we extracted three distinct neurophenotypes from multivariate neuropsychological data collected in adults recovering from SARS-CoV-2 infection. Risk factors and 6-month recovery outcomes were distinct across neurophenotypes, which provides preliminary validation of this approach. Several findings emerged that can potentially be used to guide evaluations of post-COVID patients and clinical trials of therapeutics designed to target the cognitive sequelae of long COVID.

First, most participants (69%) performed within normal limits on objective cognitive measures during the post-acute recovery stage. These participants were classified in the “normal cognition” cluster, although they did report mild severity inattention, fatigue, memory, and pain complaints. Such complaints are often sufficient to prompt evaluation in post-COVID care clinics (Graham et al., 2021), particularly if there is subjective experience of health change/decline. On average, this neurophenotype showed improvement in memory and psychomotor speed over time, although this may have been at least partially due to practice effects. Membership in this group predicted normal functional outcomes 6 months after SARS-CoV-2 infection, which is a point that can be used to counsel patients with mild post-COVID neuropsychiatric complaints who perform normally on objective cognitive testing.

Second, we found a rate of cognitive impairment (31%) among our participants that is consistent with that reported in the literature (Ceban et al., 2022; Becker et al., 2021, Pihlaja et al., 2023). Among the 31% of participants who showed cognitive impairment, there were two distinct clusters: a memory-speed impaired cluster and a dysexecutive cluster. This is consistent with the types of deficits that have been reported (Bertuccelli et al., 2022) but suggests two distinct patterns of impairment with different clinical implications.

The memory-speed impaired cluster can be considered the most severe neurophenotype. In addition to impaired performance on verbal memory, psychomotor speed, and reaction time measures, there was also subtly reduced performance on visual memory and cognitive flexibility measures. Individuals in this group reported the highest rates of subjective inattention, poor memory, and fatigue. They exhibited persistent impairment at 6-month follow-up and reported the highest rate of functional limitations and health change (decline) over the past year. Although medical comorbidities can increase the risk of more severe COVID-19 infection and contribute to overall health decline, membership in this cluster was not associated with medical comorbidity status in the 1-year leading up to infection. Rather, risk factors included higher COVID-19 symptom severity, lower vaccination rate (largely due to the lack of vaccine availability at time of infection), and the presence of anosmia during acute infection, all of which are disease-specific factors.

This raises the possibility that cognitive impairment in the memory-speed-impaired neurophenotype may be due to pathologic mechanisms directly related to SARS-CoV-2 infection. Higher disease severity in COVID-19 reflects an increased need for respiratory support, which suggests that hypoxic-ischemic damage is an important etiological factor to consider (Almeria et al., 2020; Thakur et al., 2021), especially as this is an established risk factor for memory impairment following critical illness in general (Pandharipande et al., 2013) and COVID-19 specifically (Thakur et al., 2021). Direct and indirect neuroinvasion must also be considered. Post-mortem investigations of SARS-CoV-2 infected patients have shown neural invasion and cell death through infected astrocytes (Crunfli et al., 2022) in regions that are part of the suspected neural–mucosal CNS entry route (Meinhardt et al., 2021) and are proximal to regional atrophy patterns implicated by neuroimaging of living patients, such as the piriform cortex, parahippocampal gyrus, and orbitofrontal cortex (Dondaine et al., 2022; Douaud et al., 2022), all of which are known to support memory and neuropsychiatric functions. An increasing number of studies also establish the inflammatory consequences of COVID-19 within the central nervous system (Vora et al., 2021). Biofluid biomarkers of astroglial activation (YKL-40) and pro-inflammatory cytokines (e.g., IL-1β, IL-6, IL-8, and TNF-α) distinguish cases from healthy uninfected controls (Pilotto et al., 2021), while markers of neuroaxonal loss (e.g., neurofilament light, total-tau) rise in proportion with disease severity, with higher levels identifying patients with worse outcomes at hospital discharge (Virhammar et al., 2021; Prudencio et al., 2021). Collectively, these findings suggest that post-COVID cognitive sequelae in the memory-speed impaired cluster may arise from the combined direct and indirect effects of COVID-19 infection on the brain.

Surprisingly, younger individuals had a higher risk of membership in the memory-speed impairment cluster. This has two important implications. One is that the memory impairment in this group is unlikely to reflect unmasking of an incipient age-related neurodegenerative disease. The second is that these are individuals who would be otherwise working, raising families; thus, persistent cognitive impairment in this cohort is likely to result in greater functional impairment, raising per capita and indirect costs of disability, similar to what has been documented in conventional brain injury groups (Lo et al., 2021). For these young patients, early and intensive cognitive rehabilitation efforts are essential, not just for recovery and community integration, but for minimizing the financial impact of COVID-19 infection.

The dysexecutive neurophenotype was characterized by impairment in complex attention and cognitive flexibility. This was a milder neurophenotype that showed recovery over six months in complex attention and cognitive flexibility. The base rates for impairment in complex attention dropped from 36% to 9.1% and for cognitive flexibility from 52% to 9.1%. However, attrition may have inflated improvements, as those who completed 6-month follow-up had higher baseline complex attention and cognitive flexibility than those who were lost to follow-up. Risk factors for cluster membership included COVID-nonspecific factors such as neighborhood deprivation and obesity. Participants from communities with higher ADI scores are more likely to experience systemic disadvantage, potentially manifesting as reduced access to physical and mental healthcare, food insecurity, reduced exercise opportunities, more air pollution and unsafe housing, social discrimination, and increased worry about pandemic-related factors (Abrams and Szefler, 2020; McNeely et al., 2020; Case et al., 2022). They are more likely to be concerned about the varied economic effects of the pandemic, school closures and coordination of work and childcare responsibilities, occupational exposure to the virus, access to and cost of healthcare, ability to socially distance, and concern for older family members potentially living in the same household, all stressors that could impact cognitive performance (Valdes et al., 2022; Bernardini et al., 2021). Obesity is more common in areas of lower socioeconomic status (Wang and Beydoun, 2007), which suggests that these may not be independent risk factors.

### 4.1. Treatment Considerations

Our findings emphasize differences and similarities across patients with long COVID symptoms. Post-acute neuropsychological profiles clustered into three distinct neurophenotypes, each associated with distinct risk factors and 6-month recovery outcomes. These findings can inform phenotype-specific approaches to treatment, highlighting the need for different treatment approaches rather than a “one size fits all” response to post-COVID symptoms. This is important for prudent programmatic resource allocation and financial effect modeling within medical provider teams and for minimizing out-of-pocket expenses incurred by patients. Importantly, we found that more than two-thirds of patients ascertained from a hospital registry do not have objective cognitive impairment. For many, inefficiencies in attention and executive functioning resolved within six months of infection. For the normal and dysexecutive neurophenotypes, reassurance and lifestyle counseling will likely be essential to improve long-term wellness, along with public and private health initiatives to strengthen pandemic childcare policies, employee sick time policies, and healthcare access. Cognitive Behavioral Therapy (CBT) is also likely to provide benefits for those reporting persistent anxiety, depression, insomnia, and fatigue (Adamson et al., 2020).

For the memory-speed neurophenotype, a comprehensive interdisciplinary rehabilitation approach that incorporates physical therapy, occupational therapy, nursing, and psychology may be particularly important, as demonstrated in comprehensive pain clinics (Engelberg-Cook et al., 2021; Kurklinsky et al., 2016). Realistic goal setting, activity pacing, and empowered self-management of symptoms are essential components of therapy (Skilbeck, 2022). Targeted cognitive rehabilitation in long COVID patients is effective for remediation of memory impairment (García-Molina et al., 2022). Rehabilitative therapies can focus on recovery strategies and compensatory memory strategies to attenuate frustration and facilitate adjustment to life with memory dysfunction. Individualized recommendations from cognitive rehabilitation specialists can inform accommodations to support a successful return to work, school, or community reintegration.

### 4.2. Limitations

A significant study limitation was high participant attrition. Although there were no significant differences in follow-up rates by cluster, the mild and severe neurophenotypes had smaller sample sizes than the normal cluster. Disproportionate cluster size was not predicted in advance due to the unknown nature of the disease. Results provide valuable information for prospective study planning. Larger cohorts will be necessary to obtain sufficient sample size for the dysexecutive and memory-speed impaired neurophenotypes in future longitudinal outcome investigations. An additional limitation is that we did not evaluate whether participants received formal interventions or therapeutics between post-acute and chronic assessments; therefore, we cannot attribute recovery to the “natural course” of the disease.

## Conclusion

The neurologic manifestations of long COVID present as distinct neurophenotypes with different risk factors and recovery trajectories. Future efforts should seek to replicate these neurophenotypes and their associated features in independent samples. It will be important to directly test whether the efficacy of various post-COVID therapeutics differs across neurophenotypes, given the high likelihood that different etiological factors contribute to post-COVID cognitive sequelae and influence recovery.

## Figures and Tables

**Table 1.** Neurophenotypes: associated features and risk factors.

Feature	Cluster 1 Dysexecutive (N = 31)	Cluster 2 Memory-Speed Impaired (N = 32)	Cluster 3 – Normal Cognition (N = 142)	*p-* *value*
**Age**, M (SD), y	50.06_a_ (13.12)	47.22_a_ (14.49)	56.81_b_ (13.50)	< 0.001*
**Sex**, n (%)				0.25
Female	22 (71.0%)_a_	21 (65.6%) _a_	80 (56.3%) _a_	
Male	9 (29.0%) _a_	11 (34.4%) _a_	62 (43.7%) _a_	
**Covid Variant Type**, n (%)				0.24
Initial/Alpha	14 (45.2%) _a_	21 (65.6%) _a_	63 (44.4%) _a_	
Delta	9 (29.0%) _a_	5 (15.6%) _a_	47 (33.1%) _a_	
Omicron	8 (25.8%) _a_	6 (18.8%) _a_	32 (22.5%) _a_	
**Hospitalization Status**, n (%)				0.05
Ambulatory	27 (87.1%)_a_	22 (68.8%)_a_	122 (85.9%)_a_	
Hospitalized	4 (12.9%)_a_	10 (31.3%)_a_	20 (14.1%)_a_	
**NIAID Score**, median (range)	8 (3, 8)_a_	7 (3, 8)_b_	8 (3, 8)_a_	0.006*
**Anosmia**, n (%)				0.002*
No Anosmia	19 (63.3%)_a_	9 (30.0%)_b_	87 (64.0%)_a_	
Anosmia	11 (36.7%)_a_	21 (70.0%)_b_	49 (36.0%)_a_	
**Vaccination Status**, n (%)				0.02*
Vaccine Unavailable	14 (45.2%)_a,b_	21 (65.6%)_a_	59 (42.8%)_b_	
Unvaccinated	5 (16.1%)_a,b_	4 (12.5%)_a_	8 (5.8%)_b_	
Vaccinated	12 (38.7%)_a,b_	7 (21.9%)_a_	71 (51.4%)_b_	
**Area Deprivation Index (ADI)**, median (range)	37 (4, 80)_a_	31 (8, 94)_a,b_	30 (2, 94)_b_	0.03*
**Elixhauser van-Walraven Index (EVCI)**, median (range)	0 (−4, 21)_a_	0 (−4, 31)_a_	0 (−4, 27)_a_	0.86
**History of Smoking**, n (%)				0.93
Yes	7 (25.0%) _a_	6 (20.7%) _a_	30 (22.6%) _a_	
No	21 (75.0%) _a_	23 (79.3%) _a_	103 (77.4%)_a_	
**Diabetes**, n (%)				0.71
Yes	2 (6.5%) _a_	4 (12.5%) _a_	13 (9.3%) _a_	
No	29 (93.5%) _a_	28 (87.5%) _a_	127 (90.7%) _a_	
**Hypertension**, n (%)				0.28
Yes	8 (25.8%) _a_	14 (43.8%) _a_	44 (31.4%) _a_	
No	23 (74.2%) _a_	18 (56.3%) _a_	96 (68.6%) _a_	
**Obesity**, n (%)				0.01*
Yes	9 (29.0%)_a_	7 (21.9%)_a,b_	14 (10.0%)_b_	
No	22 (71.0%)_a_	25 (78.1%)_a,b_	126 (90.0%)_b_	

National Institute of Allergy and Infectious Diseases (NIAID) severity scale: 8) Death; 7) Hospitalized, on invasive mechanical ventilation or extracorporeal membrane oxygenation (ECMO); 6) Hospitalized, on non-invasive ventilation or high flow oxygen devices; 5) Hospitalized, requiring supplemental oxygen; 4) Hospitalized, not requiring supplemental oxygen - requiring ongoing medical care (COVID-19 related or otherwise); 3) Hospitalized, not requiring supplemental oxygen - no longer requires ongoing medical care; 2) Not hospitalized, limitation on activities and/or requiring home oxygen; 1) Not hospitalized, no limitations on activities.

Area Deprivation Index (ADI): Socioeconomic disadvantage at the (US) Census Block Group neighborhood level ranging from 1 (least disadvantaged) to 100 (most disadvantaged) based on unemployment rates, poverty, education, and housing.

Elixhauser van-Walraven Index (EVCI): Summary index of 31 common chronic medical conditions abstracted from the electronic health record up to 1 year prior to PCR-positive test date.

Cluster columns not sharing subscripts indicate mean or median differs significantly at p < 0.05 as indicated by Bonferroni correction.

**Table 2.** Neurophenotypes: 6-month functional outcomes

	Cluster 1 - Dysexecutive Function (N = 11)	Cluster 2 - Memory-Speed Impaired (N = 12)	Cluster 3 – Normal Cognition (N = 76)	*p-* *value*
**Medical Outcomes Survey (MOS SF-36)**, M (SD)				
**Physical functioning**	108.09 (10.72)_a_	94.17 (13.99)_b_	105.62 (11.30)_a_	0.02
**Role functioning/physical**	109.64 (11.25)_ab_	94.50 (16.05)_a_	106.82 (14.28)_b_	0.03
**Role functioning/emotional**	106.09 (11.62)_a_	99.50 (16.19)_a_	105.61 (12.69)_a_	0.33
**Energy/fatigue**	103.18 (10.80)_ab_	89.08 (19.90)_b_	102.55 (16.57)_a_	0.03
**Emotional well-being**	104.09 (8.25)_a_	97.42 (16.00)_a_	104.97 (11.32)_a_	0.16
**Social functioning**	105.55 (11.06)_ab_	91.92 (16.58)_a_	103.32 (13.41)_b_	0.02
**Pain**	103.91 (11.20)_a_	90.42 (17.24)_b_	102.13 (12.45)_a_	0.01
**General health**	105.00 (13.01)_a_	87.58 (21.16)_b_	106.61 (16.63)_a_	0.002
**Health change**	101.36 (13.31)_a_	83.33 (14.20)_b_	94.91 (13.95)_a_	0.007
**PTSD PCL-17**, median (range)	21 (17-37)_a_	25 (18-65)_a_	20 (0-59)_a_	0.19

MOS SF-36 scores are standardized to mean of 100 (SD = 10) based on comparison to a U.S. normative reference group that ranges in age from 18-94 years (Ware and Sherbourne, 1992). Lower scores indicate greater functional disability.

The PTSD PCL-17 contains 17 items that are summed as a severity score (1 = not at all, 5 = extremely) from a range of 17 – 85, with higher scores indicating greater PTSD symptom severity. Scores greater than 29 indicate moderate to severe PTSD and cut-off scores ranging from 30-50 have been used to define PTSD in prior research studies (Gerrity et al., 2007).

Cluster columns not sharing subscripts indicate mean or median differs significantly at p < 0.05 as indicated by Bonferroni correction.
